# Supplementary material for: Complexome profiling of the Chlamydomonas psb28 mutant reveals TEF5 as an early PSII assembly factor
Source: Plant Cell. 2025 Mar 17;37(6):koaf055. doi: 10.1093/plcell/koaf055 (PMC12164590; doi:10.1093/plcell/koaf055)
Supplement: koaf055_Supplementary_Data [file koaf055_supplementary_data.zip › psb28_interactive.zip!/plots/intensity/qualityPlots/peptides/DAAK1_Cre01.g029750.t1.1.html]

### Cre01.g029750.t1.1

Trivial name: DAAK1  
SeqCoverage [median]: 8.20  
Mass: 187.57 kDa  
  
 

| Sequence | MissedCleavage | Mass [Da] | wtIntensity | muIntensity | Score |
| --- | --- | --- | --- | --- | --- |
| FFLIDGFPR | 0 | 1110.59 | 349.43 | 0.00 | 75.11 |
| SDDNADTIR | 0 | 1005.44 | 332.16 | 534.69 | 69.60 |
|  |  |  | 681.6 | 534.7 |  |
